# Final adult height of patients with childhood-onset systemic lupus erythematosus: a cross sectional analysis

**DOI:** 10.1186/s12969-018-0239-8

**Published:** 2018-04-23

**Authors:** Merav Heshin-Bekenstein, Liat Perl, Aimee O. Hersh, Emily von Scheven, Ed Yelin, Laura Trupin, Jinoos Yazdany, Erica F. Lawson

**Affiliations:** 1 Division of Pediatric Rheumatology, University of California San Francisco, Benioff Children’s Hospital, 550 16th Street, 5th Floor, San Francisco, CA 94143-0632 USA; 2 Division of Pediatric Endocrinology, University of California San Francisco, San Francisco, CA USA; 3 Division of Pediatric Rheumatology, University of Utah, Salt Lake City, UT USA; 4 Division of Rheumatology, University of California San Francisco, San Francisco, CA USA; 5 Philip R Lee Institute for Health Policy Studies, University of California, San Francisco, CA USA

**Keywords:** SLE, Childhood-onset SLE, Adult-onset SLE, Final adult height, Mid-parental target height, Growth hormone

## Abstract

**Background:**

To compare final height to mid-parental target height among adults with childhood-onset systemic lupus erythematosus (cSLE) versus adult-onset SLE (aSLE), and to evaluate the impact of age at SLE onset on final height.

**Methods:**

Data derived from the Lupus Outcomes Study, a longitudinal cohort of adults with SLE, was used for this cross-sectional analysis (*N* = 728). Participants aged 18–63 years with complete height data were included (*N* = 566) and were classified as cSLE if age at diagnosis was < 18 years (N = 72). The Tanner formula was used to calculate mid-parental target height. Multivariate linear regression was used to determine mean difference between final height and target height. Multivariate logistic regression was used to compare odds of substantially reduced final height, defined as > 2 SD below target height. Separate analyses were conducted for females and males to account for differences in timing of the pubertal growth spurt for each sex.

**Results:**

Participants with cSLE were, on average, 2.4 cm shorter than their target height (95% CI -4, − 0.7). The adjusted odds ratio (OR) for substantially reduced final height was 3.9 (95% CI + 2.0, + 7.2, *p* < 0.001) as compared to participants with aSLE. Females diagnosed between 11 and 13 years were at greatest risk for substantially reduced final height, with adjusted OR of 11.2 (95% CI + 3.4, + 36.3) as compared to participants with aSLE (*p* < 0.001).

**Conclusions:**

cSLE is associated with shorter-than-expected final height. Onset of SLE in the pubertal period, near the time of maximum linear growth, may have a particularly significant impact on final height.

## Background

Childhood-onset systemic lupus erythematosus (cSLE) is a multi-system autoimmune disease characterized by autoantibody production. It is estimated to account for 10% to 20% of all cases of systemic lupus erythematosus (SLE), with an average age at onset of 12 years [1].

cSLE is often more severe than adult-onset SLE (aSLE), with higher levels of disease activity, greater likelihood of renal and neurological involvement, lower complement levels and a more frequent need for immunosuppressive therapy [2–5]. Survival in SLE has improved dramatically, from five-year survival rates of 42–72% in the 1960’s to 95% today [6–8]. As a result, SLE in adults and children has shifted from being a predominantly fatal disease to a chronic condition [9, 10]. As life expectancy of children with SLE has increased, assessing outcomes of the disease, including the effects of therapy, has become increasingly important.

Impaired linear growth is commonly encountered in children with chronic inflammatory conditions, including SLE. These children may experience delayed onset of puberty and attenuated pubertal growth spurts, especially when the disease presents in late childhood or early adolescence [11, 12]. Poor growth may lead to short stature, and ultimately shorter-than-expected adult height, which may impact quality of life [11]. Multiple factors may contribute to the underlying pathophysiology of growth failure in chronic illness, including suboptimal nutrition, prolonged use of glucocorticoids, comorbidities and the chronic inflammatory process itself [11, 13]. Growth failure may result from suppression of the Growth Hormone (GH)-IGF-1 axis or at the level of the growth plate [11].

Significant differences between target and final adult height has been shown in systemic-onset juvenile idiopathic arthritis (sJIA), although these reports do not reflect the more recent outcomes of patients treated with biologics, which may reduce steroid use and subsequent growth impairment [11, 14]. Studies of adults with childhood-onset inflammatory bowel diseases (IBD) also reveal a difference between target and actual height [11], especially in males [15]. In a cohort of 123 patients with pediatric onset Crohn’s disease, mean adult height was 2.4 cm less than target height, and 19% of patients were more than 8 cm shorter than their target height [16].

Growth failure was added to the Modified SLICC/ACR SLE Damage Index (M-SDI) by Bandeira et al. [17] and Gutierrez-Suarez et al. [18] as a damage measure for pediatric SLE. Although Bandeira et al. documented growth failure in 15.8% at 3 years of follow up of cSLE, the percentage of participants with growth failure was smaller (7.7%) at 5 years of follow up, demonstrating the potential for catch-up growth in children. As a result, Hiraki et al. proposed final height as a preferred measure of damage in pediatric SDI [12], recognizing that only reduced final height represents an irreversible outcome, since children with cSLE and growth failure may experience significant catch-up growth once the disease is better controlled and steroid dose is tapered.

The purpose of this study was to assess final adult height in an adult cohort of patients with both cSLE and aSLE, comparing final height to target height and evaluating the impact of age of SLE onset on final height. To our knowledge, this work represents the first study of final adult height in cSLE.

## Patients and methods

### Data sources

**Fig. 1:**
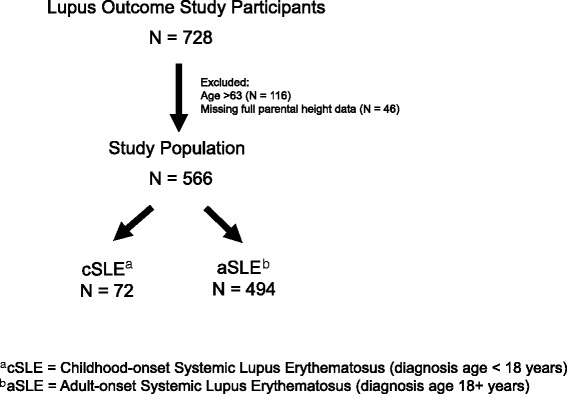
Study Population of Adults with SLE from the Lupus Outcome Study included in the Analysis

Data was derived from the 2007 cycle of Lupus Outcome Study (LOS) (Fig. 1). The LOS is a longitudinal, U.S.-based cohort of over 1200 adults with SLE, 10% of whom had disease onset in childhood (defined as age at diagnosis < 18 years). Details regarding LOS eligibility and enrollment are described elsewhere [19]. Briefly, participants were recruited from community (70%) and clinical (30%) sources, with data collected annually via telephone by trained interviewers. All participants had a confirmed diagnosis of SLE according to chart review supervised by a rheumatologist, using the American College of Rheumatology (ACR) classification criteria for SLE [20]. The survey included validated items pertaining to demographic and socioeconomic characteristics, SLE manifestations, medications, general health, mental health, cognition, employment, and health care utilization. All study data were obtained by participant self-report.

### Measures

The primary outcome measures for this analysis were 1) difference between patient-reported final adult height and mid-parental target height, and 2) substantially reduced final height. Target height was calculated from patient-reported biologic-parent heights using the Tanner formula, a validated measure used to estimate genetic height potential: mean parental height plus 6.5 cm for males, and mean parental height minus 6.5 cm for females [21]. Substantially reduced final height was defined as final adult height more than 2 standard deviations (SD) below the mid-parental target height, consistent with the clinical definition of short stature [22]. The SD for the target height in the general population is approximately 2.5 cm [23]; therefore, we identified patients with final adult height more than 5 cm below the target height as having substantially reduced final height.

The primary predictor variable was cSLE, defined as age < 18 years at diagnosis. To further explore the impact of age at SLE onset on final adult height, we sub-divided the cSLE cohort into groups based on age at disease onset (< 11, 11–13, 14–15, 16–17 years). Age groups were defined based on timing of pubertal growth spurts in healthy girls and boys. On average, the pubertal growth spurt occurs between the ages of 11 and 13 years in females and 14 to 15 years in males. Demographic predictors included sex and race/ethnicity. Race/ethnicity was categorized as White, Hispanic, African American, Asian and other. SLE-associated predictors included year of diagnosis, disease duration, steroid use ever, cyclophosphamide use ever, and history of end-stage renal disease (defined as history of dialysis or kidney transplant). Year of diagnosis was included in the final model as a predictor to account for the changes in the standard of care for SLE over time. Cyclophosphamide use and end-stage renal disease (ESRD) were included as proxies for disease severity.

### Study sample

Data from the 2007 cycle of the LOS were used for this cross-sectional analysis (*N* = 728). All participants age 18–63 years with complete height data were included (*N* = 566). Since the oldest participant with cSLE was 63 years old at time of interview, aSLE patients age > 63 years were excluded (*N* = 116) in order to maximize overlap between groups, as well as to avoid the potential confounding effect of linear height loss among elderly participants.

### Statistical analysis

Baseline characteristics of the cSLE and aSLE groups were summarized and compared using bivariate statistics (Student’s t-test, rank sum and chi-square test), as appropriate. Multivariate linear regression was used to determine adjusted and unadjusted mean differences between final adult height and target height. Multivariate logistic regression was used to compare unadjusted and adjusted odds of substantially reduced final height between participants with cSLE and aSLE. The final models included cSLE vs. aSLE status, sex, race/ethnicity and predictors of substantially reduced final height with *p* < 0.2 in univariate analysis among participants with cSLE (year of diagnosis, cyclophosphamide use and ESRD).

Among participants with cSLE, one-way ANOVA testing was used to determine whether the difference between final and target height varied according to timing of cSLE onset (age < 11, 11–13, 14–15, 16–17 years). Separate ANOVA analyses were conducted for females and males to account for differences in the average timing of the pubertal growth spurt, and post-hoc Tukey testing was conducted to account for multiple comparisons.

Bivariate and multivariate logistic regression analyses were conducted to determine whether timing of SLE onset is associated with significantly shorter-than-expected final height among female participants with cSLE. Analyses were not conducted for males alone due to the small number of male participants.

Finally, to further explore the impact of disease severity on final height among individuals with cSLE, we examined the association between substantially reduced final height and history of cyclophosphamide treatment in the cSLE cohort, using univariate and multivariate logistic regression. Covariates included sex, race/ethnicity, timing of cSLE onset (age < 11, 11–13, 14–15, 16–17 years), year of diagnosis and history of ESRD. Statistical analyses were performed using STATA 13.0 (StataCorp, College Station, TX.)

## Results

### Demographics

The study population consisted of 566 participants with SLE, including 72 (13%) with cSLE. The baseline characteristics of the cSLE and aSLE subgroups are described in Table 1. As compared to participants with aSLE, those with cSLE were younger at the time of the interview (mean age 33 ± 9 versus 49 ± 9 years; *p* < 0.001), more likely to be male (12% versus 6%; *p* = 0.01) and less likely to be white (51% versus 70%; *p* < 0.001). Mean age at diagnosis was 14 ± 3 versus 33 ± 10 years (*p* < 0.001), with ranges of age of diagnosis being 2–17 and 18–58 respectively. Respondents with cSLE were more likely to have ever required dialysis (18% versus 7%; *p* = 0.001) and to have undergone kidney transplantation (14% versus 5%; *p* = 0.003). All cSLE participants and nearly all aSLE participants reported a history of steroid use (100% versus 94%; *p* = 0.02). There was no difference between groups in the likelihood of having received cyclophosphamide (14% versus 13%; *p* = 0.9).

**Table 1:** Baseline characteristics of childhood and adult onset SLE patients enrolled in Lupus Outcome Study

Variable	cSLE^a^ (*N* = 72)	aSLE^b^(*N* = 494)	*p*-value
Sociodemographics	*N* (%) or Mean ± SD		
Age at diagnosis, years	14 ± 3	33 ± 10	< 0.001
Age at interview, years	33 ± 9	49 ± 9	< 0.001
Sex, female	63 (88%)	465 (94%)	0.01
Race/Ethnicity (%)			< 0.001
White	37 (51%)	347 (70%)	
Hispanic	11 (15%)	39 (8%)	
African American	5 (7%)	35 (7%)	
Asian	11 (15%)	50 (10%)	
Other	8 (11%)	23 (5%)	
SLE Characteristics			
Dialysis ever (A)	13 (18%)	34 (7%)	0.001
Renal transplant ever (B)	10 (14%)	24 (5%)	0.003
End-stage ever (A and/or B)	15 (21%)	37 (7%)	< 0.001
Treated w Steroids ever	72 (100%)	465 (94%)	0.02
Treated w Cyclophosphamide ever	10 (14%)	66 (13%)	0.9

^a^cSLE = Childhood-onset Systemic Lupus Erythematosus (diagnosis age < 18 years)

^b^aSLE = Adult-onset Systemic Lupus Erythematosus (diagnosis age 18+ years)

### Impact of age at SLE onset on final height

**Fig. 2:**
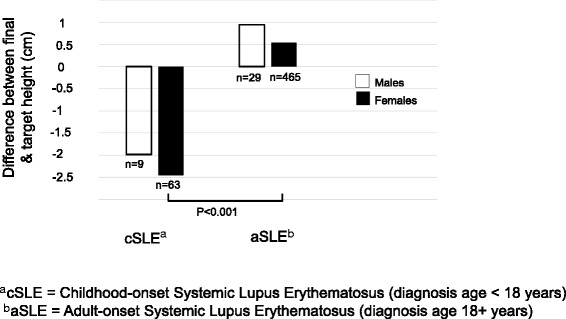
Mean difference between final height and mid-parental target height among participants with childhood-onset versus adult-onset SLE, by sex (*n* = 566). This graph demonstrates the mean difference between final height and mid-parental target height among males and females with cSLE and aSLE. Females with cSLE were on average 2.5 cm shorter than their target height (95% CI -4.3, − 0.6) which differed significantly from females with aSLE (*p* < 0.001). Males with cSLE were on average 2 cm shorter than target height, but did not differ significantly from males with aSLE (95% CI -7.5, 3.6, *p* = 0.2)

In bivariate analysis, participants with cSLE were, on average, 2.4 cm shorter than their target height (95% CI -4, − 0.7). This differed significantly from aSLE participants (*p* < 0.001), who were a mean of 0.6 cm taller than expected (95% CI 0.03, 1.1). When dividing the groups by sex, females were on average 2.5 cm shorter than their target height (95% CI -4.3, − 0.6), as compared to aSLE (*p* < 0.001). Males were 2 cm shorter than their target height, but the difference was not significant (95% CI -7.5, 3.6, *p* = 0.2) (Fig. 2). In a multivariate linear regression model controlling for sex, race/ethnicity, year of SLE diagnosis, cyclophosphamide use and ESRD, individuals with cSLE were on average 2.8 cm shorter than their target height (95% CI -4.2, − 1.3), and this also differed significantly as compared to aSLE individuals (*p* < 0.001), who were on average 0.6 cm taller than expected (95% CI 0.07, 1.2).

**Fig. 3:**
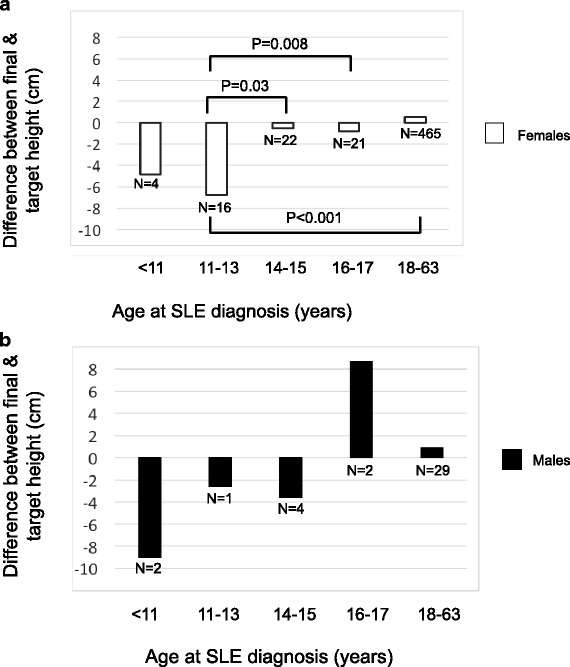
Mean difference between final height and mid-parental target height among participants by age at SLE diagnosis. **a** Mean difference between final height and mid-parental target height among females with SLE, by age at lupus diagnosis. Females diagnosed between 11 and 13 years of age were shorter than expected by 6.8 cm (95% CI -11.7, −1.8), which differed significantly from females diagnosed later in childhood (14–15, 16–17 years) or during adulthood. **b** Males diagnosed with SLE at the youngest ages (< 11, 11–13, 14–15) were on average shorter than expected, while males diagnosed at age 16–17 or in adulthood were on average taller than expected. However, these differences did not reach statistical significance

Among cSLE participants, mean difference between actual and target height differed significantly according to age at disease onset (*p* < 0.001, Fig. 3). Females with cSLE diagnosed between 11 and 13 years old were shorter than expected by 6.8 cm (95% CI -11.7, − 1.8), which differed significantly from females with aSLE (*p* < 0.001), who were on average 0.5 cm taller than expected (95% CI -0.0, + 1.1). In a multivariate linear regression model controlling for race/ethnicity, year of SLE diagnosis, cyclophosphamide use and ESRD, females with cSLE diagnosed between 11 and 13 years old were on average 8 cm shorter than their target height (95% CI -11, − 5), *p* < 0.001. Females diagnosed at 14 or 15 years old were shorter than expected by 0.5 cm, which approached statistical significance when compared to females diagnosed at 11–13 years (95% CI -3, + 2, *p* = 0.06). Males diagnosed with SLE at the youngest ages (< 11, 11–13, 14–15) were on average shorter than expected, while males diagnosed at age 16–17 or in adulthood were on average taller than expected. However, these differences did not reach statistical significance.

**Fig. 4:**
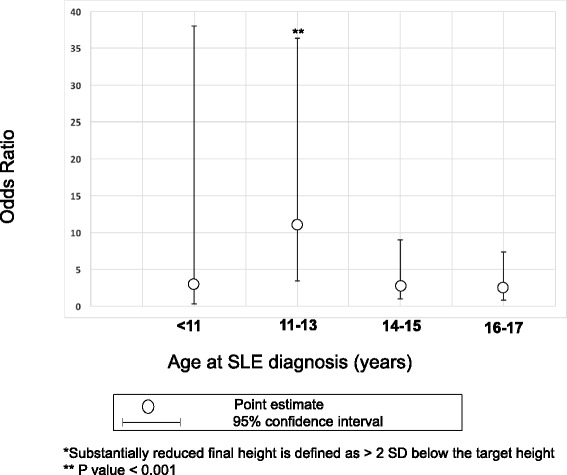
Adjusted Odds Ratios for Substantially Reduced Final Height among females with cSLE by age at diagnosis*. Females with cSLE diagnosed between the ages of 11 and 13 years were at greatest risk for substantially reduced final height (final height > 2 standard deviations below mid-parental target height), with an adjusted odds ratio of 11.2 as compared to participants with aSLE (95% CI + 3.4, + 36.3; *p* < 0.001). Analyses adjusted for race/ethnicity, year of diagnosis, cyclophosphamide use and ESRD

Finally, we compared the odds of substantially reduced final height, or actual height more than 2 SD (> 5 cm) below target height. A higher proportion of cSLE participants had substantially reduced final height when compared to the adult-onset group (31% vs 14%, *p* < 0.001). In logistic regression analyses, participants with cSLE were more likely to have substantially reduced final height, with an unadjusted OR of 2.8 (95% CI + 1.6, + 4.9, *p* < 0.001). After adjustment for sex, ethnicity/race, year of SLE diagnosis, cyclophosphamide use and ESRD, the odds of short stature in cSLE participants rose to 3.9 (95% CI + 2.0, + 7.2, *p* < 0.001). Females diagnosed between the ages of 11 and 13 years were at greatest risk for substantially reduced final height, with unadjusted odds ratio of 6.3 (95% CI + 2.3, + 17.3, *p* < 0.001) and adjusted odds ratio of 11.2 (95% CI + 3.4, + 36.3; *p* < 0.001) as compared to participants with aSLE (Fig. 4).

### Impact of cyclophosphamide on substantially reduced final height

Individuals in the cSLE cohort who received cyclophosphamide were clinically significant shorter in univariate analysis (27% vs 8%, *p* = 0.03). In multivariate logistic regression analysis accounting for sex, race/ethnicity, timing of cSLE onset (age < 11, 11–13, 14–15, 16–17 years), year of diagnosis and history of ESRD, cSLE participants with history of cyclophosphamide use were again much more likely to demonstrate substantially reduced final height, with an adjusted OR of 9.0 (95% CI + 1.5, + 53, *p* = 0.01).

## Discussion

In this cohort of adults with SLE, we found that individuals with cSLE were significantly less likely to reach their target height as compared to those with adult-onset SLE. Our results further demonstrate that onset of SLE in the pubertal period, a time of rapid linear growth, may have a particularly significant impact on final adult height.

Due to the rarity of cSLE and the lack of longitudinal cohort studies, information regarding growth and final height in cSLE has been limited. In a cross-sectional study of 1015 cSLE patients with a mean age of 15.9 years, growth failure, defined as height more than 2 standard deviations (SD) below the mean for age, was reported in 15.3% [18]. No information on target height or final adult height was included. Another cross-sectional study of 70 patients with cSLE found that mean height of the cohort was significantly lower than healthy matched controls; however, this cohort consisted of patients aged 9–49 years, and therefore did not reflect the final adult height for all patients, and did not take into account the target height for each individual [24]. In a similar study that examined 32 cSLE patients for a mean period of 4.3 years during the 1980s, short stature (defined as height for age less than the 5th percentile) was present in 38%. This study also did not include final height for all participants [25]. In a longitudinal study, the Paediatric Rheumatology International Trials Organization (PRINTO) examined growth in 331 cSLE patients with a median age of 13.9 years over 26 months, comparing patients’ height to mid-parental height [26]. At the end of the study period, growth failure (defined as a parental height z-score < − 1.5) was seen in 15% of the female patients and 25% of the male patients. Furthermore, growth failure was seen in 22.4% of women with disease onset before the age of 12 compared to 3.3% of women with disease onset after 12 years of age. Again, final adult height was not assessed.

Timing of the pubertal growth spurt is different in males and in females. The pubertal growth spurt in females occurs during early adolescence. The peak linear growth velocity corresponds most closely with Tanner stage 2 in females, which occurs in 95% of the girls by the age of 12.1 years. For males, the growth spurt occurs later in puberty, around Tanner stage 3 and age 14 years [23]. The differences between females and males in our analysis can be partially explained by this difference in timing of the pubertal growth spurt. In our analysis, SLE onset in females around the age of peak linear growth (11–13 years old) was associated with significantly shorter than expected adult height, whereas SLE diagnosed later in puberty (age 14–15 years and 16–17 years), after the growth spurt is completed for most females, did not significantly affect final adult height. Males demonstrated similar results, though these findings did not achieve statistical significance, likely due to the very small number of male participants in our study. Males who were diagnosed around the time of peak linear growth (age 14–15 years) were shorter than expected by a mean of 3.6 cm. Disease onset between ages 11 to 13 years in males did affect final height as well, but to a lesser extent than in the female population.

Final height greater than 2 SD below the mid-parental height (> 5 cm) has been previously suggested by Hiraki et al. as a damage measure in cSLE [12]. In our analyses, individuals with cSLE were more than three times more likely to meet this criterion as compared to the aSLE population. Odds of short stature were the greatest among females diagnosed between the ages of 11–13 years.

Our analyses demonstrated that childhood-onset lupus patients with a history of cyclophosphamide exposure may be at significantly higher risk for reduced final height. As individuals with cSLE who receive cyclophosphamide typically have severe disease, they are likely to have had higher levels of systemic inflammation and steroid exposure, both of which can inhibit linear growth.

Although to date recombinant GH therapy is not indicated in the treatment of chronic inflammatory disease, cSLE patients with significant short stature may be considered for GH therapy in the future. In other inflammatory diseases like juvenile idiopathic arthritis, randomized controlled trials have confirmed that GH therapy can improve final adult height and also suggest a modest effect on short to medium term catch-up growth [11]. The benefit versus risk from GH therapy should be considered, with the main risks being decrease in insulin sensitivity, skeletal complications like avascular necrosis, increased risk of malignancies and the theoretical risk of worsening the inflammatory disease process itself. In the literature, there are only case reports of GH therapy in cSLE. Gorska et al. reports on GH therapy given to a 17-year-old female patient with short stature and bone age of 11 years [27]. Following one-year of GH therapy, the girl achieved catch-up growth, with no significant side effects. Bae et al. reported a case of a 19-year-old man with a history of cSLE, treated with GH and testosterone at the age of 16 for hypogonadism and growth impairment [28]. Nine months after GH initiation, height increased by 10 cm. However, GH therapy was discontinued due to increased disease activity, which improved after cessation of GH therapy. Yap et al. described improved growth velocity after initiation of supra-physiological doses of GH in a 15-year-old male with cSLE, though the patient subsequently developed worsening of lupus nephritis, which improved following cessation of the GH treatment [29].

There are several limitations to our study. First, since the height data in our cohort relies on participant self-report, inaccuracies may occur. However, self- and family member- reporting of heights are widely used in the literature, and have been proven accurate in the adult population [30, 31]. Second, since the LOS collected data from individuals with cSLE in adulthood, we do not have detailed data on exposures or outcomes that occurred during childhood, such as cumulative steroid exposure, disease activity measurements, nutritional status or financial status. Since we lack information on cumulative steroid exposure or steroid dosing during childhood, the present study cannot assess whether the effect of steroids on the final height is dose-dependent, or whether there is a dose that minimizes the risk of shorter-than-expected final height. Despite the absence of disease activity measurements at the time of cSLE diagnosis, it can be assumed that SLE is active at the time of diagnosis. Therefore, if SLE was diagnosed at the time of puberty, lupus activity and treatment may impede the pubertal growth spurt, ultimately limiting adult height. It is important to note that we do not have data on the exact timing of puberty relative to SLE diagnosis, e.g. age at menarche or Tanner stage at diagnosis. Therefore, we used age as a proxy for the timing of the pubertal growth spurt. However, age may not accurately reflect pubertal stage, especially among adolescents with cSLE that may be at risk for delayed puberty. Similarly, we do not have data to specify the timing of other events that occurred in cSLE participants, such as the onset of ESRD during childhood vs. adulthood. Third, the number of male participants is our study was small, which reflects the fact that SLE is much more common in females. This reduced our power to detect statistically significant differences among male participants. Finally, it is important to note that our cSLE cohort is on average younger than the aSLE cohort. Since younger generations have become progressively taller over the past century [32], as reflected by the fact that our aSLE cohort was on average taller than mid-parental target height, the true target height of our cSLE cohort may actually be greater than their mid-parental height. Therefore, the true difference in the odds of clinically significant short stature between our aSLE and cSLE cohorts may be greater than reported here.

This study is the first to assess final adult height in individuals with childhood-onset SLE, which is a more accurate indicator of permanent damage than growth failure. Our study also included data on mid-parental height, which allowed us to account for the impact of genetics in a multi-ethnic cohort.

Our study suggests that disease onset at the period of maximum linear growth may predict shorter-than-expected stature in adulthood. Per our results, clinicians should closely monitor growth in patients with pubertal onset of SLE and consider underlying pubertal development in their determination of future risk for short stature. Treatment strategies that minimize steroid exposure and promote tight disease control, including aggressive use of second-line immunosuppressive agents, should be applied when possible, as minimizing systemic inflammation and steroid exposure may help to prevent short stature in cSLE. Individuals with severe short stature may benefit from GH therapy; however, further data is needed in order to determine risk versus benefit and to identify the sub-groups for whom this treatment is most appropriate. The development of predictive models for future short stature would be informed by prospective studies that assess all potential risk factors for reduced final height, including detailed assessments of disease features, medication exposure and pubertal stage.

## Conclusion

In conclusion, our study showed that childhood-onset SLE is associated with shorter-than-expected final adult height. Onset of SLE in the pubertal period, a time of rapid linear growth, may have a particularly significant impact on final adult height.

## Abbreviations

aSLE: Adult-onset Systemic Lupus Erythematosus
cSLE: Childhood-onset Systemic Lupus Erythematosus
GH: Growth Hormone
LOS: Lupus Outcome Study
SLE: systemic lupus erythematosus
